# Prevalence of epilepsy and seizure history in Finnish neuropathologically examined medico-legal autopsy cases with acute head injuries

**DOI:** 10.1186/s13104-026-07894-4

**Published:** 2026-06-02

**Authors:** Gaia Narayan, Petteri Oura

**Affiliations:** 1 Department of Forensic Medicine, University of Helsinki, P.O. Box 63, FI-00014 Helsinki, Finland; 2 Forensic Medicine Unit, Finnish Institute for Health and Welfare, P.O. Box 30, FI- 00271 Helsinki, Finland

**Keywords:** Forensic pathology, Epilepsy, Seizure, Traumatic brain injury, Central nervous system

## Abstract

**Objectives:**

Epilepsy and traumatic brain injury have a bidirectional relationship. This study aimed to report the prevalences of antemortem epilepsy diagnosis, seizure history, epilepsy-related and traumatic neuropathological findings, as well as background characteristics, among neuropathologically examined medico-legal autopsy cases with acute head injuries.

**Results:**

The sample included 82 cases from Helsinki, Finland, from the years 2016—2022. Of the sample, 17 cases had an epilepsy diagnosis or documented seizure history (“Epilepsy/Seizure Group”), whereas 65 did not (“No Seizure Group”). The most frequent epilepsy-related neuropathological findings were hippocampal atrophy and mossy fibre sprouting. Of background characteristics, the Epilepsy/Seizure Group had a higher prevalence of old head injuries (41.2% vs. 7.7%) and chronic alcohol use (41.2% vs. 18.5%) than the No Seizure Group. There were no statistically significant differences in acute head injuries or secondary neuropathological findings, except for a lower prevalence of oedema in the Epilepsy/Seizure Group (17.6% vs. 47.7%). Even though the Epilepsy/Seizure Group had a higher prevalence of old head injuries and chronic alcohol use, the prevalences of acute head injuries were not higher. Larger studies are needed to further explore whether individuals with epilepsy or seizure history are susceptible to distinct head injury types or circumstances.

## Introduction

The International League Against Epilepsy defines epileptic seizure as “transient occurrence of signs and/or symptoms due to abnormal excessive or synchronous neuronal activity in the brain” [1]. Epilepsy is defined as “pathologic and enduring tendency to have recurrent seizures”; not all seizures constitute a diagnosis of epilepsy. Epilepsy affects over 50 million people worldwide [2]. Its aetiology is diverse and includes entities such as traumatic brain injury (TBI), cerebrovascular complications, tumours, infections, perinatal insults, malformations of cortical development, metabolic disorders, hippocampal sclerosis, and neurodegeneration [3]. Notably, posttraumatic epilepsy is estimated to account for 10–20% of all epilepsy cases [4]. As individuals with epilepsy are at an increased risk of sudden death [5], as well as unnatural death [6, 7], the entity is highly relevant in medico-legal cause-of-death investigation.

Of specific brain regions, hippocampus and cerebral cortex are among the areas commonly associated with epilepsy [8, 9]. Hippocampal sclerosis is characterised by neuronal loss and gliosis as well as granule cell reorganization [10]. Mossy fibre sprouting (i.e., axonal penetration into the molecular layer of the dentate gyrus) is another hippocampal finding among cases with epilepsy, often associated with sclerosis [10]. As for the cortex, malformations of cortical development (e.g., focal cortical dysplasia, heterotopia, and polymicrogyria) are relatively common causes of focal epilepsy [11]. A wide range of other pathological lesions within the intracranial space may also be considered epileptogenic [3].

The association of TBI with seizures and epilepsy has been shown in various studies [4, 12, 13]. In the event of mechanical trauma to the brain, damage may prompt a neuroinflammatory reaction which may predispose to seizure activity [14]. Importantly, a bidirectional relationship between epilepsy and TBI has been observed, as pre-existing epilepsy may increase the risk of sustaining severe and repetitive TBI [15, 16], even more so than drug or alcohol abuse [17] and particularly in accident scenarios [18]. However, there is a paucity of studies addressing the prevalence of epilepsy, seizure history, and associated neuropathological findings in acute TBI.

The aims of this study were two-fold. The first aim was to describe the prevalences of antemortem epilepsy diagnosis, seizure history, and epilepsy-related neuropathological findings, among neuropathologically examined medico-legal autopsy cases with acute head injuries. The second aim was to compare the types of primary head injuries, secondary neuropathological findings, and background characteristics among the cases with and without an antemortem epilepsy diagnosis or seizure history. We hypothesised that epilepsy/seizure cases would entail a higher prevalence of old head injuries, as well as higher prevalences of primary head injuries and secondary findings, compared to the negative cases.

## Material and methods

### Material

According to the Finnish law, deaths that are sudden and unexpected, and those that involve a suspicion of accident, homicide, suicide, medical or surgical adverse event, occupational disease, or war, are referred to the police for a cause-of-death investigation. These deaths are generally subjected to a medico-legal autopsy that is performed by a forensic pathologist in the Forensic Medicine Unit, Finnish Institute for Health and Welfare. Approximately 15% of all deaths are subjected to a medico-legal autopsy in Finland [19, 20].

This study was based on medico-legal autopsy cases from the Helsinki office of the Forensic Medicine Unit between the years 2016 and 2022. The Helsinki office is responsible for performing medico-legal autopsies for deaths that occur in the greater capital region and Southern Finland, with over 3000 autopsies annually [21]. The dataset included all medico-legal autopsy cases that met the following criteria: (1) an acute head injury was identified, and (2) the forensic pathologist referred the formaldehyde-fixed brain to a neuropathological examination. Neuropathological examinations were performed by board-certified neuropathologists. Acute head injuries could be identified in either the general autopsy or neuropathological examination.

An electronic information system of the Forensic Medicine Unit (“OLT”) was used to retrieve the official case files and obtain the necessary variables for the analysis. Among other data, the system contains information related to medico-legal cause-of-death investigation, including clinical data on medical history.

The procedures were in accordance with the ethical standards of the Helsinki Declaration. Ethical approval and informed consent were not required, as this was a register-based study. Research permit to use the data was obtained from the Finnish Institute for Health and Welfare (THL/1802/6.02.00/2023).

### Epilepsy-related variables

Cause-of-death investigation documents were queried for (1) an antemortem diagnosis of epilepsy, together with its most likely aetiology, and (2) an antemortem suspicion of epilepsy, based on documented recurrent seizures but lack of epilepsy diagnosis. No distinction was made regarding the presence or absence of provoking factors, as this information was not consistently available.

The following case groups were formed:

- Cases with an antemortem epilepsy diagnosis or documented seizure history (”Epilepsy/Seizure Group”).
- Cases with an antemortem epilepsy diagnosis (”Epilepsy-Only Subgroup”), constituting a subgroup within the Epilepsy/Seizure Group.
- Cases with no antemortem epilepsy diagnosis or documented seizure history (”No Seizure Group”).

The neuropathologist’s report was queried for the following epilepsy-related neuropathological findings: hippocampal atrophy (i.e., reduced size and/or neuronal loss of the hippocampus), together with its severity (mild/moderate/sclerosis [i.e., significant neuronal loss and gliosis in the hippocampal formation]) and location (right/left/bilateral); hippocampal mossy fibre sprouting (i.e., axonal penetration into the molecular layer of dentate gyrus); and other potential epileptogenic lesions.

### Background variables

Cause-of-death investigation documents were queried for the following background variables: sex of the decedent (male/female); age at death (years); general circumstance of the injury that preceded death (fall/traffic accident/assault/unknown or other); chronic alcohol use (i.e., antemortem diagnosis of alcohol use disorder, or records of chronic excessive alcohol consumption); and old head injuries.

In addition, the following autopsy variables were collected: body height (cm), body weight (kg), and fresh brain weight (g). Postmortem interval was calculated based on the number of days between the death and autopsy.

The manner of death (disease/accident/homicide/other) was obtained from the death certificate that was issued by a forensic pathologist at the end of the cause-of-death investigation. Causes of death were recorded according to the 10th revision of the International Statistical Classification of Diseases and Related Health Problems (ICD-10). Causes were categorized in a data-driven manner into groups with at least five cases each: head or neck injury (S00-S19, T04.0, T06.0), asphyxia (T17, T71, T75.1), intoxication (T36-T65), cardiovascular disease (I10-I52), epilepsy (G40-G41, inclusive of all subcodes), stroke (I60-I69), and other.

### Head injury variables and secondary findings

The medico-legal autopsy report and neuropathologist’s report yielded data on the presence of acute head injuries: scalp haemorrhage; skull fracture; traumatic epidural, subdural, subarachnoid, or intracerebral haemorrhage; contusion; and traumatic axonal injury based on β-amyloid precursor protein stain. Moreover, the following secondary neuropathological findings were recorded: brain oedema, either macroscopic or microscopic; vascular axonal injury in β-amyloid precursor protein stain; and hypoxic-ischaemic neuronal injury (i.e., neuronal eosinophilia and pyknosis in haematoxylin-eosin stained samples from frontal and parietal cortical watershed regions, hippocampus, lenticular nucleus, thalamus, brainstem, and cerebellum).

### Statistical analysis

SPSS Statistics version 27 (IBM, Armonk, NY, USA) was used to analyse the data. Descriptive statistics included percentages and frequencies for categorical variables, and medians and interquartile ranges (IQRs) for continuous variables. First, the prevalences of epilepsy-related neuropathological findings were calculated for the Epilepsy/Seizure Group and the Epilepsy-Only Subgroup. Then, distributions of background characteristics, and prevalences of primary head injuries and secondary neuropathological findings were calculated for the following groups: full sample, Epilepsy/Seizure Group, Epilepsy-Only Subgroup, and No Seizure Group.

As for comparative analyses, two sets of statistical comparisons were performed. Primarily, the Epilepsy/Seizure Group was compared to the No Seizure Group, and secondarily, the Epilepsy-Only Subgroup was compared to the No Seizure Group. The Epilepsy-Only Subgroup was evaluated as a sensitivity analysis, excluding cases with seizure history but no epilepsy diagnosis. Between-group comparisons were performed using non-parametric tests (i.e., Fisher Exact Test for binary variables, Fisher-Freeman-Halton Test for variables with > 2 classes, and Mann-Whitney U Test for continuous variables), since some of the subgroups were small. The threshold for statistical significance was *P* < 0.05.

## Results

The dataset comprised 82 neuropathologically examined medico-legal autopsy cases with acute head injuries. The median postmortem interval was 6 (IQR 3–9) days. Of the sample, a total of 12 cases (14.6%) had an antemortem diagnosis of epilepsy; aetiologies were old TBI (*n* = 4), old spontaneous intracerebral haemorrhage (*n* = 2), lysosomal storage disorder (*n* = 2), old infarct (*n* = 1), and unknown (*n* = 3). An additional 5 cases (6.1%) had an antemortem suspicion of epilepsy based on documented recurrent seizures, but diagnostic investigations were still in progress at the time of death.

The cases with an antemortem epilepsy diagnosis or documented seizure history are collectively referred to as the Epilepsy/Seizure Group (*n* = 17), and the negative cases are referred to as the No Seizure Group (*n* = 65). As a subgroup within the Epilepsy/Seizure Group, the cases with an antemortem epilepsy diagnosis are referred to as the Epilepsy-Only Subgroup (*n* = 12).

Table 1 presents a summary of epilepsy-related neuropathological findings among the Epilepsy/Seizure Group and the Epilepsy-Only Subgroup. Hippocampal atrophy and/or mossy fibre sprouting were reported among 6 cases. In addition, a vascular malformation (*n* = 1) and signs of old contusion, intracerebral haemorrhage, and infarct (*n* = 2 each) were observed as epileptogenic lesions. In two cases, the neuropathological findings were consistent with a previously diagnosed storage disorder. All findings were observed in the Epilepsy-Only Subgroup, i.e., among cases with a pre-existing antemortem epilepsy diagnosis.

Background characteristics, injury circumstances, and causes of death are summarized in Table 2. The majority of the sample comprised male decedents (69.5%) and the median age was 49 (IQR 27–71) years, with no statistically significant differences between the groups. However, chronic alcohol use was statistically significantly more common among the Epilepsy/Seizure Group (41.2%) than among the No Seizure Group (18.5%). Records of old head injury were also statistically significantly more common among the Epilepsy/Seizure Group (41.2%) than the No Seizure Group (7.7%). When the Epilepsy-Only Subgroup was compared to the No Seizure Group, the difference in old head injuries persisted (41.7% vs. 7.7%), whereas the difference in chronic alcohol use was not statistically significant (33.3% vs. 18.5%).

**Table 1:** Prevalence of epilepsy-related neuropathological findings among cases with an antemortem epilepsy diagnosis or documented seizure history (Epilepsy/Seizure Group), and among the cases with an antemortem epilepsy diagnosis (Epilepsy-Only Subgroup)

Finding	Epilepsy/Seizure group (*n* = 17)	Epilepsy-Only Subgroup (*n* = 12)
Hippocampal atrophy	35.3 (6)	50.0 (6)
Side		
Right	5.9 (1)	8.3 (1)
Left	11.8 (2)	16.7 (2)
Bilateral	17.6 (3)	25.0 (3)
Severity		
Mild	23.5 (4)	33.3 (4)
Sclerosis	11.8 (2)	16.7 (2)
Hippocampal mossy fibre sprouting	29.4 (5)	41.7 (5)
Vascular malformation	5.9 (1)	8.3 (1)
Old lesion		
Contusion	11.8 (2)	16.7 (2)
Intracerebral haemorrhage	11.8 (2)	16.7 (2)
Infarct	11.8 (2)	16.7 (2)
Storage disorder	11.8 (2)	16.7 (2)

Numbers are percentages with frequencies

**Table 2:** Background characteristics of the sample. Breakdown of cases with an antemortem epilepsy diagnosis or documented seizure history (Epilepsy/Seizure Group), cases with an antemortem epilepsy diagnosis (Epilepsy-Only Subgroup), and cases without seizure history (No Seizure Group)

Characteristic	Full sample (*n* = 82)	Epilepsy/Seizure Group (*n* = 17)	Epilepsy-Only Subgroup (*n* = 12)	No Seizure Group (*n* = 65)
General characteristics				
Male sex	69.5 (57)	76.5 (13)	66.7 (8)	67.7 (44)
Age (y)	49 (27–71)	43 (28–57)	42 (20–70)	49 (27–73)
Body height (cm)	172 (159–178)	173 (162–184)	169 (157–175)	172 (156–178)
Body weight (kg)	70 (55–84)	76 (65–98)*	73 (62–87)	64 (54–82)
Brain weight (g)	1414 (1283–1531)	1374 (1332–1537)	1371 (1329–1511)	1424 (1279–1525)
Record of chronic alcohol use	23.2 (19)	41.2 (7)*	33.3 (4)	18.5 (12)
Record of old head injury	14.6 (12)	41.2 (7)*	41.7 (5)*	7.7 (5)
Circumstance of recent head injury				
Fall	19.5 (16)	11.8 (2)	16.7 (2)	21.5 (14)
Traffic accident	4.9 (4)	5.9 (1)	0.0 (0)	4.6 (3)
Assault	26.8 (22)	29.4 (5)	33.3 (4)	26.2 (17)
Unknown/other	48.8 (40)	52.9 (9)	50.0 (6)	47.7 (31)
Manner of death				
Disease	45.1 (37)	47.1 (8)	50.0 (6)	44.6 (29)
Accident	19.5 (16)	17.6 (3)	16.7 (2)	20.0 (13)
Homicide	19.5 (16)	17.6 (3)	25.0 (3)	20.0 (13)
Other	15.9 (13)	17.6 (3)	8.3 (1)	15.4 (10)
Cause of death				
Head or neck injury	20.7 (17)	17.6 (3)	16.7 (2)	21.5 (14)
Asphyxia	9.8 (8)	5.9 (1)	8.3 (1)	10.8 (7)
Intoxication	9.8 (8)	5.9 (1)	0.0 (0)	10.8 (7)
Cardiovascular disease	8.5 (7)	5.9 (1)	0.0 (0)	9.2 (6)
Epilepsy	6.1 (5)	29.4 (5)*	41.7 (5)*	0.0 (0)
Stroke	6.1 (5)	0.0 (0)	0.0 (0)	7.7 (5)
Other	39.0 (32)	35.3 (6)	33.3 (4)	40.0 (26)

Numbers are percentages with frequencies or medians with interquartile ranges, as appropriate

*P < 0.05 in comparison to the No Seizure Group (Fisher‘ s Exact Test, Fisher-Freeman-Halton Test, or Mann-Whitney U Test, as appropriate)

Regarding the injury circumstances preceding death, unknown or other circumstances were the most common in all groups, followed by assaults (Table 2). Manners of death also had similar distributions in all groups; most deaths were natural, followed by other manners. There were no statistically significant differences in the cause-of-death distributions, apart from the fact that 29.4% of the Epilepsy/Seizure Group and 41.7% of the Epilepsy-Only Subgroup had epilepsy as their underlying cause of death (vs. 0% in the No Seizure Group). Of the cases whose cause of death was epilepsy (*n* = 5), two died of status epilepticus (witnessed or strongly suspected) and three had sudden unexpected death in epilepsy (SUDEP; definite or possible).

A breakdown of acute head injuries and secondary findings is presented in Table 3. The commonest injuries were scalp haemorrhage, subarachnoid haemorrhage, and traumatic axonal injury among all groups. Even though most injuries had somewhat higher prevalences among the No Seizure Group than the Epilepsy/Seizure Group or Epilepsy-Only Subgroup, there were no statistically significant differences in individual injury types between the groups. As for secondary findings, oedema was significantly more prevalent among the No Seizure Group than the Epilepsy/Seizure Group (47.7 vs. 17.6%); the difference between the Epilepsy-Only Subgroup and the No Seizure Group was not statistically significant.

**Table 3:** Primary head injuries and secondary neuropathological findings among sample. Breakdown of cases with an antemortem epilepsy diagnosis or documented seizure history (Epilepsy/Seizure Group), cases with an antemortem epilepsy diagnosis (Epilepsy-Only Subgroup), and cases without seizure history (No Seizure Group)

Finding	Full sample (*n* = 82)	Epilepsy/Seizure Group (*n* = 17)	Epilepsy-Only Subgroup (*n* = 12)	No Seizure Group (*n* = 65)
Primary head injury types				
Scalp haemorrhage	74.4 (61)	58.8 (10)	58.3 (7)	78.5 (51)
Skull fracture	11.0 (9)	5.9 (1)	8.3 (1)	12.3 (8)
Epidural haemorrhage	1.2 (1)	0.0 (0)	0.0 (0)	1.5 (1)
Subdural haemorrhage	9.8 (8)	5.9 (1)	8.3 (1)	10.8 (7)
Subarachnoid haemorrhage	37.8 (31)	35.3 (6)	33.3 (4)	38.5 (25)
Intracerebral haemorrhage	3.7 (3)	0.0 (0)	0.0 (0)	4.6 (3)
Contusion	7.3 (6)	0.0 (0)	0.0 (0)	9.2 (6)
Traumatic axonal injury	23.2 (19)	23.5 (4)	16.7 (2)	23.1 (15)
Secondary findings				
Oedema	41.5 (34)	17.6 (3)*	16.7 (2)	47.7 (31)
Vascular axonal injury	18.3 (15)	11.8 (2)	16.7 (2)	20.0 (13)
Hypoxic-ischaemic neuronal injury	79.3 (65)	88.2 (15)	83.3 (10)	76.9 (50)

Numbers are percentages with frequencies

*P < 0.05 in comparison to the No Seizure Group (Fisher‘s Exact Test)

## Discussion

This study of 82 neuropathologically examined medico-legal autopsy cases with acute head injuries identified only a few background characteristics and neuropathological findings that differed between the cases with an epilepsy diagnosis or seizure history and those with no seizure history. As for background characteristics, records of old head injuries and chronic alcohol use were significantly higher in the Epilepsy/Seizure Group. In contrast, the prevalences of acute head injuries and secondary neuropathological findings were not higher among cases with an epilepsy diagnosis or seizure history; instead, oedema was less prevalent in the Epilepsy/Seizure Group than in the No Seizure Group.

In this dataset, the prevalences of antemortem epilepsy diagnosis (14.6%) and documented seizure history without an epilepsy diagnosis (6.1%) were clearly higher than in the general population [2]. Even though the high prevalence may be partly explained by a higher rate of neuropathology consultations among cases with an epilepsy diagnosis or seizure history, this also suggests that epilepsy presents in a relatively frequent concomitance with acute TBI. Of note is the paucity of previous literature on the prevalence of epilepsy diagnoses and epilepsy-related findings in medico-legal autopsy cases with acute head injuries.

Of the cases with an antemortem epilepsy diagnosis, the most common aetiology was TBI (33.3%), followed by unknown causes (25.0%), old haemorrhagic (16.7%) and ischaemic lesions (8.3%), and metabolic disorders (16.7%). As shown in previous literature, epilepsy may be triggered by a number of factors [3], and the aetiological diversity was present also in the present dataset. The high prevalence of posttraumatic epilepsy was in line with previous reports [4].

The present dataset revealed a statistically significant association between epilepsy and history of head injuries, which aligns with previous literature [4, 12, 13]. Previous studies have suggested that TBI triggers neuroinflammation and hypoxic-ischaemic damage that may lead to epilepsy [14, 22]. Pre-existing epilepsy increases the risk for circumstances in which TBI may occur, partly due to the impact seizures have on daily life and autonomic control [15, 16]. This projects as an elevated risk for unnatural death [6, 7]. Moreover, the Epilepsy/Seizure Group had a high prevalence of chronic alcohol use (41.2%); not surprigingly, alcohol use is associated with both TBI and seizures [23]. It can be speculated that some individuals with seizures but no epilepsy diagnosis, as well as some individuals with epilepsy diagnosis, may have had alcohol-related seizures.

No statistically significant differences were found in injury circumstances and primary head injuries between cases with and without epilepsy or seizure history. In absolute terms, the No Seizure Group showed higher prevalences of nearly all primary head injuries than the other groups. Although moderate sample size reduced statistical power, our findings did not appear to support the initial hypothesis that primary head injuries would be more common in cases with epilepsy or seizure history. A previous Finnish study had similar findings, reporting high prevalences of intracranial haemorrhages in fatal TBI cases, regardless of epilepsy status [24]. Epilepsy was associated mostly with falls among the elderly, but there were no circumstantial or injury-specific variables of statistical significance in the study. Further research is needed to explore whether individuals with epilepsy or seizure history are susceptible to distinct head injury types or circumstances.

As for secondary neuropathological findings, the prevalence of oedema was clearly highest in the No Seizure Group. Considering that the absolute prevalences of nearly all primary head injuries were higher in the No Seizure Group, and that head and neck injuries were most frequently set as the cause of death among the No Seizure Group, there may have been differences in the overall severity of TBI between the groups. As epilepsy was assigned the primary cause of death in some cases (rather than TBI itself), neuropathological examination likely had a role in excluding fatal injuries and other lesions, thus contributing to the cause-of-death investigation. In any case, these data do not support our hypothesis of more extensive head injuries among cases with epilepsy or seizure history.

The strengths of this study include the systematic inclusion of all neuropathologically examined cases with acute head injuries that underwent medico-legal autopsy at our office over the study period. The neuropathological examinations were performed comprehensively by board-certified neuropathologists. A relatively large selection of variables could be addressed in the study, covering both background characteristics and neuropathology findings. Although there is previous literature on the association between epilepsy and old/recurrent TBI [4, 12], postmortem studies have rarely addressed the prevalence of epilepsy and neuropathological findings in cases with acute head injury. Our study contributes by providing a breakdown of these characteristics in a medico-legal autopsy sample.

In conclusion, among Finnish neuropathologically examined medico-legal autopsy cases with acute head injuries, the prevalence of antemortem epilepsy diagnosis was 14.6%, and the commonest aetiology was TBI. The prevalence of documented seizure history without an epilepsy diagnosis was 6.1%. Of epilepsy-related neuropathological findings, hippocampal atrophy and mossy fibre sprouting were the most frequent ones. Of background characteristics, records of old head injuries and chronic alcohol use were more common among cases with than without epilepsy or seizure history. There were no statistically significant differences supporting the hypothesis that acute head injuries or secondary neuropathological findings would be more prevalent among cases with epilepsy. We expect knowledge on head injury susceptibility in epilepsy to be relevant to both forensic pathologists and clinicians.

## Limitations

First, all autopsy cases with acute head injuries were not consistently referred to a neuropathological examination, as this decision was made by the forensic pathologists on a case-by-case basis. To provide contextual data, the total number of medico-legal autopsy cases in which a head injury (corresponding to ICD-10 codes S00-S09, T04.0, T06.0) was recorded as the cause or factor contributing to death over the study period was 1233. As the present sample comprised all 82 neuropathologically examined cases with acute head injuries, it seems that most cases were managed without a neuropathology consultation. This somewhat reduced the representativeness of the present sample relative to all medico-legal autopsy cases. Similarly, as the sample only included cases with acute head injuries, the results are not directly generalisable to living populations with epilepsy. Second, our sample size was moderate, which reduced the statistical power of the analysis. Third, we made no distinction between unprovoked and provoked seizures, as their exact nature and underlying circumstances were not always described comprehensively. Fourth, our dataset did not include specific mechanisms of the recent head injuries, and the severity or accumulation of primary head injuries were not addressed. Fifth, our dataset exhibits heterogeneity, and the groups may well differ with respect to underlying unmeasured variables. As we performed crude comparisons between groups, it was not possible to draw conclusions about confounding or causality.

## Data Availability

The datasets analyzed during the study are not made public due to local privacy regulations. Access to the dataset can be obtained from the Finnish Institute for Health and Welfare, with permission (Finnish Social and Health Data Permit Authority, https://findata.fi/en/).
